# Sensory Profiles of Children with Autism Spectrum Disorder with and without Feeding Problems: A Comparative Study in Sicilian Subjects

**DOI:** 10.3390/brainsci10060336

**Published:** 2020-05-31

**Authors:** Simonetta Panerai, Raffaele Ferri, Valentina Catania, Marinella Zingale, Daniela Ruccella, Donatella Gelardi, Daniela Fasciana, Maurizio Elia

**Affiliations:** 1 Oasi Research Institute-IRCCS, 94018 Troina, Italy; rferri@oasi.en.it (R.F.); vacatania@oasi.en.it (V.C.); mzingale@oasi.en.it (M.Z.); dgelardi@oasi.en.it (D.G.); melia@oasi.en.it (M.E.); 2 Psychoeducational Service for Children with Autism and Intellectual Disability, Società Cooperativa Sociale “I Corrieri dell’Oasi” (CdO), 94100 Enna, Italy; danielaruccella82@gmail.com; 3 Center for Diagnosis and Early Intensive Treatment of Autism Spectrum Disorder, 93100 ASP Caltanissetta, Italy; danifasciana@gmail.com

**Keywords:** autism spectrum disorder (ASD), sensory profile, sensory responsiveness, feeding problems, short sensory profile (SSP), sensory experience questionnaire (SEQ)

## Abstract

The aim of this study is to better understand the relationship between sensory and feeding problems in Autism Spectrum Disorder (ASD) by comparing sensory responsiveness of ASD children with (ASD-W) and without (ASD-WO) feeding problems. The feeding and sensory characteristics of 111 children with ASD (37 ASD-W and 74 ASD-WO) were assessed by using two questionnaires tapping on feeding problems and two on sensory problems. A comparative study was carried out with between-group as well as intra-group comparisons design; a correlation analysis was also added. A statistically significant correlation was found between sensory and feeding problems. ASD-W children showed more severe and extensively impaired sensory responses than ASD-WO, with lower sensory adaptation and more generalized and severe deficits in all subdomains. Taste/Smell sensitivity was strongly impaired only in ASD-W, whereas in ASD-WO it was found to be a point of strength. Both groups showed a Hyporesponsive profile, though it was more marked in ASD-W. Both groups showed strengths in Visual/Auditory sensitivity, Low-Energy/Weak, and Movement sensitivity, again more marked in ASD-WO. These results might prove to be particularly useful for sensory training and psychoeducational treatment.

## 1. Introduction

Sensory impairments are frequent in children with Autism Spectrum Disorder (ASD) [1,2,3,4,5,6,7], with over 90% of cases presenting severe sensory symptoms in multiple sensory domains, as reported in some studies [2,4]. They form a group of disorders that involve challenges in modulation, integration, organization, and discrimination of sensory inputs, leading to either inappropriately responding to those inputs or experiencing disruptions in daily activities and emotional-behavioral patterns. In particular, sensory modulation disorders are classified into three subtypes: (1) over-responsivity (or hyperresponsiveness), characterized by exaggerated, rapid onset and/or prolonged reactions to sensory stimulation; (2) under-responsivity (or hyporesponsiveness), with unawareness or slow response to sensory input; and (3) seeking for, involving craving of, and interest in sensory experiences that are prolonged or intense [8]. Some patterns, such as hypo- and hyperresponsiveness, are also known to co-occur in children with ASD [9,10,11,12], especially in children showing a generalized sensory impairment [13]. Results of a meta-analysis by Ben Sasson et al. [14] showed a significantly high difference between ASD and Typically Developing (TD) groups in sensory modulation, with the greatest difference in under-responsivity, followed by over-responsivity and sensation seeking. Although the three sensory modulation disorder subtypes are hypothetical, some physiological research supports these distinctions. A review by Suarez [4] reported some results of these physiological studies: in over-responsivity a low threshold for one or multiple sensation channels has been hypothesized, resulting, for example, in exaggerated reactions to textures or noises. On the contrary, in under-responsivity, a high threshold has been hypothesized, so that only intense and sustained stimuli can obtain attention by children, resulting in diminished or no response, for example, to name or pain. Studies using physiological tools, such as electrodermal sensors or cardiac vagal tone index, found a decreased or increased activation of the electrodermal responses (in under-responsive and in over-responsive children respectively) and an impaired less effective parasympathetic system.

The most affected sensory modalities in children with ASD seem to be auditory filtering and tactile sensitivity [3,15,16,17,18]. Sensory dysfunctions also seem to be related to the severity of ASD [5,14,19] and to stereotyped interests and behaviors [16,20]. Limited and stereotyped behaviors, interests and activities can be observed also in the feeding domain [21,22] and a strong sensitivity to sensory information has been associated with feeding problems, especially food fussiness, in both children with typical and atypical development [23]. However, in children with atypical development, feeding concerns still persist beyond childhood. With age, children with typical development change their appetite, food preferences, and eating habits, but the social and emotional dimensions of food remain stable and expand over time. Furthermore, in children with ASD, feeding involves both the nutritional and the emotional-relational dimensions. The term “eating problems” typically refers to oral consumption of nutrients that deviates from the norm, enough to lead to negative emotional, social or health consequences. The prevalence of feeding problems in children with ASD is estimated to be approximately 90%, with 70% of children showing food selectivity [24,25,26]. It has been suggested that feeding problems might be related to sensory modulation disorders as well [4,27,28,29,30]. The study by Zobel-Lachiusa et al. [28] investigated differences in feeding behaviors and sensory characteristics of children with ASD compared to TD children. Statistically significant differences between the two groups were found in all the measures administered, as well as a moderate to strong positive correlation between feeding problems and sensory impairments in children with ASD. Nadon et al. [29] found a definite and probable difference in sensory processing (as measured by using the Short Sensory Profile—SSP) [31] in 65% of children (*N* = 95) with ASD, aged 3 to 10 years; these results were also related to increased feeding problems in the sample. Chistol et al. [30] found higher levels of food refusal in ASD children with atypical oral sensitivity compared to those showing a typical oral sensitivity. McCormick et al. [7] showed no significant differences between ASD and Developmentally Delayed children, except for the Taste/Smell and Visual/Auditory sensitivity (SSP subsections).

Children with ASD present with higher sensory problems than TD, with a prevalence ranging from 65.3% to 84.8% [3,28,32]; moreover, sensory and feeding problems seem to be correlated. Objective of our study was to investigate whether ASD children with feeding problems (ASD-W) show the same sensorimotor features as the ASD without feeding problems (ASD-WO): which sensory modalities are impaired and to what extent? Our study is not directed to feeding per se, as a purely physiological process, but to the behavioral components accompanying the feeding act.

Our hypothesis is that sensory profiles might present different characteristics in specific subgroups of children with autism (with and without feeding problems), and that ASD-W children might show higher sensory impairments than ASD-WO, with Taste/Smell sensitivity mostly affected. In order to carry out this assessment, we used four measures: two questionnaires for the detection of feeding problems, and two for the detection of sensorimotor features. We are not aware of previous studies in the literature on such a topic. A better understanding of the association between feeding problems and sensory factors in children with autism, might bring benefits for the physical health of ASD children, since it might provide some ideas and guide for treatment: for example, feeding training might include behavioral techniques to gradually decrease the negative sensory experiences related to food consumption, and encourage familiarity and acceptance of foods.

## 2. Materials and Methods

### 2.1. Study Design

A comparative study was carried out. The four questionnaires were administered by clinical psychologists, working in the diagnostic services of three Sicilian specialized centers, throughout interviews to parents, as part of the psychological and psychoeducational assessment. Recruitment and organization of the sample are described in the following paragraph.

### 2.2. Participants

A total of 111 children with ASD, aged 2 to 12 years (86 males and 25 females; median chronological age 62 months, interquartile range 44–75 months), were consecutively recruited from specialized services of diagnosis and treatment of autism during the year 2019, in three specialized centers of Eastern Sicily provinces (Enna, Caltanissetta and Catania). All participants were diagnosed by a multidisciplinary team, following the DSM-5 criteria [21]. The severity of their disorders was classified into three levels (1 to 3) accordingly. Approximately 65% (*N* = 72) of children showed a severity level of 3, about 27% (*N* = 30) a severity level of 2, and about 8% (*N* = 9) a severity level of 1; moreover, the majority of them (91%, *N* = 101) presented with comorbid Intellectual Disability (ID). Diagnoses were further confirmed using at least one of the most common diagnostic scales (the Autism Diagnostic Interview-Revised, the Autism Diagnostic Observation Schedules or the Childhood Autism Rating Scale-Second edition). Based on the results obtained at the Brief Autism Mealtime Behaviors Inventory (BAMBI-18) [33], the sample was divided into two subgroups, which included ASD-W (*N* = 37, 33%; scores at BAMBI ≥ 34) and ASD-WO (*N* = 74, 67%; scores at BAMBI < 34) children. The characteristics of the two subgroups are shown in Table 1 (*N* of males and females, level of severity, chronological ages, total and sub-domain scores obtained at mealtime behavior measures).

### 2.3. Measures

BAMBI 18 [33,34] is an 18-item interview for assessing mealtime behavior problems. A 5-point Likert scale is used, ranging from 1 = never/rarely to 5 = always, including a neutral midpoint; a total frequency score is derived from the sum of the items; higher scores indicate more problematic mealtime behaviors. Undesirable behaviors can be analyzed also on the basis of four main factors, and namely: limited variety/food selectivity, disruptive mealtime behaviors, food refusal and mealtime rigidity [34]. Original test-retest reliability was reported at 0.87 (TD children *N* = 40 and ASD children *N* = 68, aged 3 to 11 years), and interrater reliability at 0.78 [31]. The scale internal consistency was 0.88 (Cronbach’s alpha). A cut-off total score of 34 was found by DeMand et al. [34].

CEBQ [35] is a tool for assessing children’s eating styles. It is an interview including 35 items; a 5-point Likert scale is used, ranging from 1 = never to 5 = always. It includes eight scales: Food responsiveness, Emotional over-eating, Enjoyment of food, Desire to drink, Satiety responsiveness, Slowness in eating, Emotional under-eating, and Food fussiness. Internal reliability, derived from two samples (*N* = 177 and *N* = 222, respectively) for the eight factors, ranged from 0.72 to 0.91 (Cronbach’s alpha). Test-retest reliability (*N* = 160) ranged from 0.52 to 0.87.

SSP [31] is a 38-item caregiver questionnaire, scored on a 5-point Likert scale (ranging from 1 = always to 5 = never). The lower the score, the more atypical sensory responses. The SSP consists of a total score and 7 subsection scores: Tactile sensitivity (7 items, mostly focusing on tactile avoiding and expression of distress: for example, fights or cries during hair cutting, fingernail cutting or face washing; emotional or aggressive reactions to touch; difficulty in standing in line or close to other people; avoids going barefoot, especially over sand or grass); Taste/Smell sensitivity (4 items, focusing on food avoiding and selectivity: for example, avoiding certain tastes or food smells that are typically part of children’s diet; eating only certain foods or limiting to particular food textures/temperatures; picky eating); Movement sensitivity (3 items, focusing on anxiety due to specific postures: for example, becoming anxious or distressed when feet leave the ground, disliking activities where head is upside down); Under-responsive/Seeks sensation (7 items, focusing on actions adding more intense sensations and inattention during social interactions: for example, seeking to make noises, or enjoying strange noises; fidgeting, and not seating still; seeking all kinds of movement, touching people and objects, jumping from one activity to another, not noticing messy face or hands); Auditory Filtering (6 items, focusing on distraction and inattention caused by auditory stimuli in the environment: for example, being distracted by noises, and impossibility to work with background noises; not hearing what he/she is told, not responding to his/her name, ignoring persons interacting with him/her); Low Energy/Weak (6 items, focusing on weakness and easy fatigue: for example, getting easily tired, especially when standing or holding particular body positions; weak grasping; not lifting heavy objects in comparison to children with the same age); Visual/Auditory sensitivity (5 items, focusing on negative responses to unexpected or loud stimuli: for example, negative responses to unexpected or loud noises, protecting ears from sound or eyes from light, being bothered by bright light). The SSP total score and the subsection scores can be used to classify children’s sensory impairments into three categories: Typical Performance, Probable Difference, and Definite Difference. Cut-off points to define these categories are available for the total and the subsection scores. A discriminant validity > 95% in differentiating children with and without sensory impairments was found [36]. The internal reliability was reported as ranging between 0.70 to 0.90. Internal validity correlations for the subsections ranged from 0.25 to 0.76 and were all significant at *p* < 0.01 [31].

The Sensory Experience Questionnaire (SEQ Version 1) [10,37] is a brief caregiver interview designed to assess sensory problems in young children with ASD and related Developmental Disorders. A 5-point Likert scale is used, ranging from 1 = almost never to 5 = almost always. Higher scores indicate higher sensory problems. The SEQ measures the Hyper- and Hyporesponsive patterns across social and nonsocial contexts. Hypo- is considered as a lack of or delayed response to sensory stimuli [14,38]). Hyper- is defined as an exaggerated or avoidant response to sensory stimuli [39,40]. SEQ yields both a total score as well as four-dimensional subscale scores. The psychometric properties of SEQ were evaluated by Little et al. [35] by means of 358 caregiver questionnaires; internal consistency and test–retest reliability were 0.80 (Cronbach’s coefficient alpha); intraclass correlation coefficients was 0.92.

### 2.4. Statistical Analysis

Non-parametric statistics were used because most of the variables did not show a normal distribution, based on asymmetry and kurtosis. The between-group comparisons were carried out by means of the Mann-Whitney’s U test; the significance level was set at *p* < 0.05. Effect sizes were calculated by means of the *r* = *z*/√*N* formula, where N is the total number of the sample participants. The *r* value of 0.1, 0.3 and 0.5 indicated a small, a medium, and a large effect size respectively; the absolute value of r has been reported. The Chi-square test was used for frequency data, including the intra-group comparisons; effect size was calculated by means of the Cramer’s V test, where scores ≤0.2 indicate a small effect size, while scores between 0.2 and ≤ 0.6 a moderate effect size, and >0.6 a strong effect size. For intra-group comparisons, the Friedman and the Wilcoxon matched pair tests were used. Effect sizes were calculated by means of *r* = *z*/√*N* formula. A correlation analysis between sensory and feeding problems was carried out for the whole sample, by using the Spearman’s test.

### 2.5. Ethics Committee Approval

Approval was obtained from the Local Ethics Committee “Comitato Etico IRCCS Sicilia–Oasi Maria SS.”, approval date 7 July 2018, approval code: 2018/07/18/CE-IRCCS-OASI/14. All parents provided written informed consent prior to the administration of the questionnaires.

## 3. Results

No significant differences were found between ASD-W and ASD-WO subgroups, neither in the chronological ages, nor in the number of male and female participants, or in the levels of disorder severity (Table 1); therefore, the two subgroups were comparable.

Table 1 shows statistically significant differences at BAMBI and CEBQ questionnaires, both investigating feeding problems. In all the BAMBI subdomains (selectivity, disruptive behaviors, refusal, and mealtime rigidity), statistically significant differences were obtained with medium to large effect sizes. In the CEBQ, the statistically significant differences were found only in some subdomains, namely enjoyment of food, satiety responsiveness, slowness in eating, and, above all, food fussiness, including food selectivity items. Therefore, the two subgroups turned out to be clearly distinguished from each another as for feeding behaviors.

### 3.1. Comparisons Between ASD-W and ASD-WO Subgroups

Statistically significant differences were found in the comparisons between ASD-W and ASD-WO subgroups (Mann Whitney’s U test; Table 2).

As far as the SSP results are concerned, differences were found in the total score, with a medium effect size, and in almost all the subsections, with a large effect size in Taste/Smell sensitivity, a medium effect size in Auditory Filtering and Under-responsive/Seeks sensation, and a small effect size in Tactile sensitivity, Low Energy/Weak, and Movement sensitivity. Higher sensory impairments turned out to characterize the ASD-W subgroup. No differences were found in the Visual/Auditory sensitivity.

Statistically significant differences emerged from SEQ, both in the total score, with a small effect size, and in the Hyperresponsiveness subsection (in total score and responsiveness toward social and non-social stimuli), with most impairments in the ASD-W subgroup. No statistically significant differences were found in Hyporesponsiveness subsection, impaired in both subgroups (see median scores). Table 3 shows the SSP performance categories (Definite Difference, Probable Difference, and Typical Performance in both total and subsection scores), expressed in percentages of children, and the results from the comparisons between the two subgroups (Chi Square test).

The percentage of Definite Difference in ASD-W subgroup was higher than ASD-WO in both the SSP total score and the subsections; on the contrary, higher percentages of ASD-WO children fell into the Typical Performance of SSP total and subsection scores. Statistically significant differences were found in the comparisons between the two subgroups, with moderate effect sizes in the total score and in all the subsections, except for Visual/Auditory sensitivity performance, in which no statistically significant difference was found. When comparing Definite Differences vs. Typical performance, statistically significant differences and moderate effect sizes were found.

### 3.2. ASD-WO Intra-Group Analysis

**Figure 1:**
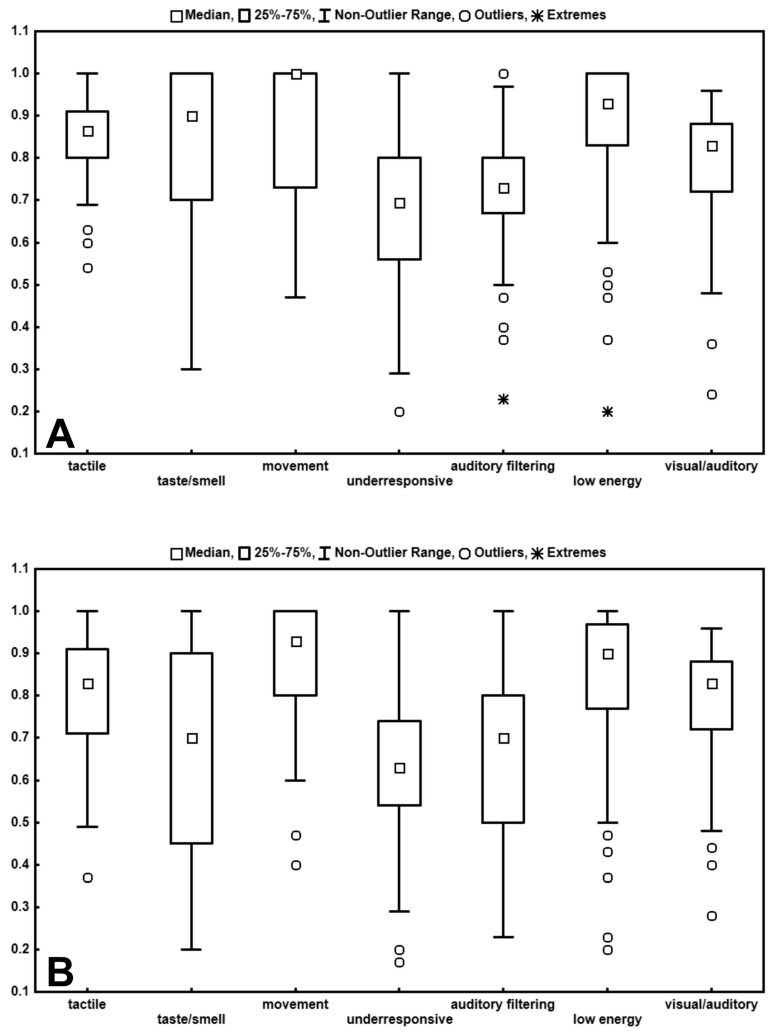
Scores obtained from ASD-WO (**A**) and ASD-W (**B**) in the SSP subdomains (expressed as medians of the ratios between obtained scores and maximum possible scores).

In the ASD-WO subgroup, a statistically significant difference (*p* < 0.00001; Friedman’s test) was found between the SSP subsection scores, expressed as medians of the ratios between the scores obtained and the maximum possible scores; the medians of the ratios ranged from 0.8 to 1, (the highest SSP score, the lowest impairment) in the following subdomains: Tactile, Taste/Smell, Movement, Visual/Auditory sensitivity and Low Energy/Weak; and from 0.7 to 0.75 in Under-responsive/Seeks sensation and Auditory Filtering (Figure 1A).

Both Under-responsive/Seeks sensation (US) and Auditory Filtering (AF) showed statistically significant differences (Wilcoxon matched pairs test) when compared to the other SSP subdomains, and namely: US vs. Tactile, Movement, and Low Energy *p* < 0.000001, with large effect sizes (*z* = 6.78, 6.45, 5.98 respectively; *r* = 0.79, 0.75, 0.69 respectively); US vs. Visual/Auditory *p* < 0.000002, with large effect size (*z* = 4.73; *r* = 0.55); US vs. Taste/Smell *p* = 0.000014, with large effect size (*z* = 4.35; *r* = 0.506); US vs. AF *p* = 0.012, with small effect size (*z* = 2.51; *r* = 0.29); AF vs. Tactile, Movement, Low Energy *p* < 0.000001, with large effect sizes (*z* = 5.76, 5.53, 5.98 respectively; *r* = 0.67, 0.64, 0.69 respectively); AF vs. Taste/Smell *p* = 0.001, with medium effect size (*z* = 3.27; *r* = 0.38), AF vs. Visual/Auditory *p* = 0.002, with medium effect size (*z* = 3.1; *r* = 0.36).

As far as the SEQ is concerned, a statistically significant difference with large effect size between the Hyperresponsiveness and Hyporesponsiveness subdomains (Wilcoxon matched pairs test, *p* < 0.00001, *z* = −5.22, *r* = 0.07) was found, with higher scores in Hyporesponsiveness. Within the Hyporesponsiveness subdomain, a statistically significant difference with medium effect size was found between responses to social and non-social stimuli (Wilcoxon matched pairs test, *p* = 0.006, *z* = 2.73, *r* = 0.036), with response to non-social stimuli being more impaired. Within the Hyperresponsiveness subdomain, no significant difference was found between responses to social and non-social stimuli.

### 3.3. ASD-W Intra-Group Analysis

In the ASD-W subgroup, a statistically significant difference (*p* < 0.00001, Friedman’s test) was found between the SSP subsection scores, expressed as medians of the ratios between the scores obtained and the maximum possible scores. The medians of the ratios ranged from 0.75 to 0.9 in the following subdomains: Tactile, Movement, Visual/Auditory and Low Energy/Weak; from 0.53 and 0.45 in Auditory Filtering, Under-responsive/Seeks sensation, and Taste/Smell sensitivity (Figure 1 B). No statistically significant differences were found either between Taste/Smell and both Under-responsive and Auditory Filtering, or between Under-responsive and Auditory filtering (Wilcoxon matched pairs test). Taste/Smell, Under-responsive and Auditory Filtering subsections showed statistically significant differences and large effect sizes in comparison with the others SSP subdomains, and namely: Taste/Smell (TS) vs. Tactile *p* = 0.000004, *z* = 4.62, *r* = 0.76; TS vs. Movement *p* = 0.000003, *z* = 4.71, *r* = 0.77; TS vs. Low Energy *p* = 0.000016, *z* = 4.32, *r* = 0.71; TS vs. Visual/Auditory *p* = 0.000017, *z* = 4.31, *r* = 0.71; US vs. Tactile *p* = 0.000002, *z* = 4.8, *r* = 0.79; US vs. Movement and Visual/Auditory *p* = 0.000001, *z* = 5.1 and 4.82 respectively; *r* = 0.84 and 0.79 respectively; US vs. Low Energy *p* = 0.000007, *z* = 4.51, *r* = 0.74 (Wilcoxon matched pairs test).

As far as the SEQ is concerned, a statistically significant difference between the Hyperresponsiveness and Hyporesponsiveness subdomains and a large effect size (Wilcoxon matched pairs test *p* <0.015, *z* = −3.39, *r* = 0.09) were found, with sensory Hyporesponsiveness being prevalent; no statistically significant differences were found either in Hyperresponsiveness or Hyporesponsiveness subdomains (responses to social and non-social stimuli).

### 3.4. Correlation Analysis

Statistically significant correlations (Spearman’s test; *p* < 0.05) between the total scores of sensory and mealtime behaviors questionnaires were found (BAMBI vs. CEBQ = 0.61; SSP vs. SEQ = −0.59; SSP vs. BAMBI = −0.46; SSP vs. CEBQ = −0.61; SEQ vs. BAMBI = 0.3; SEQ vs. CEBQ = 0.33). A positive correlation was found between the two questionnaires on feeding problems (increased scores in one questionnaire corresponded to increased scores in the other), and between SEQ and each of the mealtime measures (increasing scores in feeding problems corresponded to increasing scores in sensory impairments, investigated by SEQ). A negative correlation was found between each mealtime measure and SSP: increasing scores in BAMBI or CEBQ corresponded to decreasing scores in SSP, that is, decreased typical sensory performances.

## 4. Discussion

Children with ASD seem to show more sensory problems than children with TD, and a relationship between sensory and feeding problems was hypothesized by some authors [4,26,27,28,29]. To the best of our knowledge, no studies on ASD sensory processing have so far reported any comparisons between sensory characteristics of ASD children with and without feeding problems. The aim of our study was to shed light on this aspect, starting from the hypothesis that ASD-W children presented with higher sensory impairments than ASD-WO, with Taste/Smell sensitivity particularly affected.

In our ASD sample, the percentage of feeding problems turned out to be lower than those reported in other studies [24,25,41,42], probably due to the specific cut-off used in our study for classifying children with feeding problems. This result should be further investigated in future studies with larger samples.

The Spearman’s test showed a statistically significant correlation between feeding behaviors and responses to sensory stimuli, especially between SSP and the two mealtime behaviors measures, thus confirming the findings of the studies above mentioned [4,27,28,29,30].

Results obtained from the between-group comparisons seem to confirm our hypothesis. Statistically significant differences in the total scores of both SSP and SEQ were found, with higher impairments in the ASD-W subgroup. As stated by O’Donnell et al. [43], the SSP total score is considered as the most sensitive indicator of sensory dysfunction. As for the SSP subsections, differences turned out to be particularly marked in the Taste/Smell sensitivity, and this is certainly consistent with our expectations, since the Taste/Smell sensitivity is a factor that strongly influences feeding behaviors. The presence of olfactory and gustatory dysfunctions in children with ASD have already been reported in the literature [44,45], even if they remain still understudied. The olfactory system is mainly implicated in detection, identification, memory and recognition of odors. The principal function of taste is to analyze chemosensory, somesthetic and hedonic features of gustatory stimuli. Results from the few studies in which physiological measures were used, found significant differences in odor and taste identification in children with ASD compared with TD, but not in detection thresholds, thus suggesting cortical rather than brainstem dysfunction [45]. A recent study by Koehler et al. [46] on odor threshold and odor identification in children with ASD, using structural magnetic resonance imaging, found decreased threshold and identification functions, and a decreased activation of the pyriform cortex, suggesting that olfactory impairments in people with ASD has its correspondence in the primary olfactory cortex. A study by Avery et al. [47] on taste reactivity in ASD found an aberrant function of the primary gustatory cortex (anterior insula and frontal operculum) and other brain regions associated with social functioning. Taste and smell cerebral pathways present some similarities, since they both involve regions such as the orbitofrontal cortex, the insula, the limbic system and the hypothalamus. However, studies have so far found no clear link between impaired responses to sensory stimuli and the underlying brain sensory processing; therefore, the unusual sensory responses in children with autism still need a reliable explanation. We know that these cortex areas are connected not only to the olfactory and gustatory systems, but also to emotions and social behaviors; therefore, it is possible to hypothesize that the altered perception of taste/smell stimuli in children with autism can contribute to their social and emotional deficits.

As far as the other results of our study are concerned, statistically significant differences were found in all the other SSP subsections, especially in Auditory Filtering and Under-responsive/Seeks sensation (Table 2 and Table 3), and also in Tactile sensitivity, Movement sensitivity and Low Energy/Weak (Table 3). These results indicate that feeding behaviors of children with autism seem to be affected not only by Taste/Smell sensitivity, but also by multiple sensory experiences, and that a generalized impairment of all sensory modalities occurs more in children with ASD-W than in children with ASD-WO. This generalized sensory impairment, especially in children with ASD-W, might find an explanation in the theory of imbalance in excitatory and inhibitory processes in the brain, resulting in increased excitation (greater glutamatergic signaling) and reduced inhibition (reduced GABA inhibitory neurotransmitters) [48,49]. Increased activation was found in children with ASD in primary sensory cortices, amygdala, hippocampus and orbitofrontal cortex, also related to cognitive, emotional, and social processing; reduced GABA levels were found in auditory, motor and frontal cortex. However, a link between changes in neurotransmitters and the anomalous behavioral responses to sensory stimuli has not been established yet.

The presence and severity of sensory dysfunctions in our sample seemed to be related to feeding problems, rather than to the autism severity (as stated by some authors) [7,15,18]; in fact, the two subgroups did not differ from each other as for the severity of autistic symptoms (see Table 1). Visual/Auditory sensitivity did not seem to affect feeding behaviors: a high percentage of children (about 60% of ASD-W and 70% of ASD-WO) showed a Typical Performance in this sensory modality, therefore it was not widely impaired in our whole sample.

In a study by Lane et al. [50], using the SSP, four distinct sensory subtypes were identified: sensory adaptive, consisting in typical functioning in five of the seven SSP subsections; taste smell sensitive, consisting in extreme Taste/Smell sensitivity and clinically significant concerns in Auditory Filtering and Under-responsive/Seeks sensation; postural inattentive, consisting in extreme score in Low Energy/Weak and clinically significant concerns in Auditory Filtering and Under-responsive/Seeks sensation; and generalized sensory difference, in which all sensory domains were affected. We applied these subtypes to both ASD-W and ASD-WO children, thus confirming our results as described above: ASD-W children fell into the Taste/Smell sensitive subtype, and showed a more generalized sensory difference than the ASD-WO subgroup; on the contrary, ASD-WO children were sensory adaptive in about half of the cases.

The SSP performance classifications in our study appeared to be different from those obtained in previous studies [3,29,32] (Table 3), either in the whole ASD sample, or in the ASD-WO subgroup. In fact, the percentages of Definite Differences were lower, while those of Typical Performance higher; our percentages were close to those of the studies mentioned above only in the case of the ASD-W subgroup. This might be due to the fact that these studies included ASD children with and without feeding problems, but their percentages were not specified. Nadon et al. [29], however, suggested that the presence of feeding problems might have affected the results of their study.

As far as the SEQ is concerned, Hyporesponsiveness turned out to be impaired in both ASD-W and ASD-WO children. This is consistent with previous studies [14] and confirms data obtained in the SSP Under-responsive/Seeks sensation subsection. Statistically significant differences were found by comparing the two subgroups, both in the SEQ total score and Hyperresponsive subsection, and ASD-W appeared to be more affected than ASD-WO children; however, the effect sizes were small, therefore these results need to be confirmed by further studies. In the ASD-W subgroup the dysfunctioning seemed to be more generalized, because no differences were found in the responses to social and non-social stimuli in Hyporesponsiveness.

Results from the intra-group SSP analysis (Figure 1) are also consistent with those described above: in the ASD-WO children, the Under-responsive/Seeks sensations and Auditory Filtering subsections appeared to be the most impaired, unlike Tactile, Taste/Smell, Movement, Low Energy/Weak, and Visual-Auditory subsections, which turned out to be points of strength. The strengths of the ASD-W subgroup seem to be Movement, Low Energy/Weak, and Visual/Auditory sensitivity, unlike Under-responsive/Seeks sensation, Auditory Filtering, and Taste/Smell subsections, which turned out to be strongly impaired.

Results from our study might offer some suggestions about the opportunity to include individualized sensory training within the psychoeducational treatments. Whereas these trainings seem to be useful for ASD-WO children with no typical performance, they become essential for children with ASD-W, because of their generalized sensory dysfunctioning that requires a multisensory stimulation treatment. The multisensory stimulation treatment should be individually designed to decrease the functional limitations due to the impaired sensory modulation and should involve active participation of children to produce adaptive responses. A play context might encourage children participation. Individualized sensory trainings might provide multiple benefits: (1) normalizing the deficient responses to tactile, olfactory and tasting stimuli, so as to provide the opportunity for learning adequate mealtime behaviors and other functional self-care skills; (2) providing the appropriate required level of sensory stimulation—through Applied Behavior Analysis procedures—that would replace sensations typically experienced from repetitive behaviors or stereotypies; (3) capturing and maintaining attention on the provided stimuli (especially auditory stimuli); (4) using visual/auditory and kinesthetic stimulation (in which the ASD-W children appear to be more adapted) as a bridge to more easily accept sensory stimuli generally avoided.

Our study has some limitations: first of all, the absence of a TD control group. The comparison with TD peers would highlight any statistically significant differences with ASD sensory profiles, especially for the ASD-WO subjects, who showed more adaptive sensory responsiveness. Second, the absence of other clinical control groups, for example peers with ID, with and without feeding problems, that might allow us to better understand whether different clinical populations would show different sensory profiles. To this regard, for example, McCormick et al. [7] did not find any differences between ASD and Developmentally Delayed children in the SSP total scores, except for Taste/Smell and Visual/Auditory sensitivities. Third, the relatively small sample size, especially the ASD-W subgroup: a future study with larger samples might confirm the similarities and differences found here between ASD-W and ASD-WO sensory profiles; moreover, larger samples might be useful to establish whether changes in the sensory processing occur across different age ranges. Finally, our sample mainly included children with moderate to severe disorder (predominantly showing severity levels 2 and 3), therefore our results might be not generalizable to ASD children with a severity level 1. Fourth, to investigate children’s response to sensory stimuli we used only questionnaires to parents, that present a certain degree of subjectivity. More psychophysical investigations are needed in order to decrease the subjectivity of data, although the biological mechanisms underlying sensory processing are not easy to determine due to the heterogeneity of the disorder and the difficulty in applying physiological measures to low functioning and little collaborating subjects.

## 5. Conclusions

We were able to confirm our original hypothesis of the existence of a significant correlation between sensory and feeding problems in ASD. We may suggest that ASD-W children show a more severe and more extensive impaired sensory processing than ASD-WO children, and that feeding behaviors of children with autism are affected not only by Taste/Smell, but also by multiple sensory experiences. ASD-W and ASD-WO subgroups showed a Hyporesponsive profile. Both subgroups showed impairments in Under-responsive/Seeks sensation and Auditory Filtering, more marked in ASD-W than in ASD-WO children. Taste/Smell sensitivity was strongly impaired only in ASD-W, whereas in ASD-WO subgroup it turned out to be a strength. ASD-W showed a more severe impairment also in Tactile sensitivity. Both subgroups showed strengths in Visual/Auditory sensitivity, Low-Energy/Weak, and Movement sensitivity, though more marked in ASD-WO. Based on our results, some useful suggestions on sensory trainings and psychoeducational treatments might be provided. However, a deeper knowledge of sensory dysfunctions in larger samples of ASD-W and ASD-WO children would be desirable for better orienting psychoeducational treatments by providing pointily suggestions about specific and individualized sensory trainings.

## Figures and Tables

**Table 1:** Characteristics of the sample and scores obtained at BAMBI 18 and CEBQ, expressed as medians and interquartile ranges.

TitleSample Features	ASD-W	ASD-WO	*z* =	*p* ≤	Effect Size ^3^ *r*
*N* =	37	74			
Males/Females	30/7	56/18		NS ^1^	
Severity level 3/2/1	27/8/2	45/22/7		NS ^1^	
Chronological age, months	60 (44–76)	63.5 (45.2–74.0)		NS ^2^	
BAMBI 18 total scores	41 (38–46)	26.5 (22.25–29.75)	−8.44	0.00001 ^2^	0.8
BAMBI 18 subdomains					
Food Selectivity	14 (12–16)	9 (7–11)	−7.1	0.00001 ^2^	0.67
Disruptive Behaviors	13 (10–16)	7.5 (6–9)	−6.48	0.00001 ^2^	0.61
Food Refusal	7 (5–9)	4 (3–4.75)	−7.02	0.00001 ^2^	0.67
Mealtime Rigidity	8 (6–11)	4 (3–7)	−5.69	0.00001 ^2^	0.54
CEBQ	93 (84–105)	10 (7–16)	−8.54	0.00001 ^2^	0.81
CEBQ subdomains					
Food Responsiveness	12 (7–16)	10 (7–16)	−0.424	NS ^2^	
Emotional Over-eating	5 (4–8)	6 (4–7)	0.237	NS ^2^	
Enjoyment of food	13 (10–16)	16 (13–17)	2.939	0.038 ^2^	0.28
Desire to Drink	7 (5–9)	6 (5–8)	−0.88	NS ^2^	
Satiety responsiveness	12 (10–15)	11 (8–13)	−2.36	0.018 ^2^	0.22
Slowness in Eating	12 (10–15)	9 (8–12)	−2.13	0.033 ^2^	0.2
Emotional Under-eating	10 (6–12)	8 (6–12)	−1.84	NS ^2^	
Food Fussiness	21 (18–26)	16 (12–19)	−4.58	0.00001 ^2^	0.435

ASD = Autism Spectrum Disorder; ASD-W = ASD With feeding problems; ASD-WO = ASD WithOut feeding problems; Severity level: 3 = requiring very substantial support, 2 = requiring substantial support, 1 = requiring support; BAMBI = Brief Autism Mealtime Behaviour Inventory; CEBQ = Child Eating Behaviour Questionnaire; NS = Not Significant; ^1^ Chi-square test; ^2^ Mann-Whitney’s U Test; ^3^ Effect size calculated by using *r*= *z*/√*N* formula.

**Table 2:** Scores obtained from ASD-W and ASD-WO subgroups at the SSP and SEQ, expressed as medians and interquartile ranges.

MeasuresTitle	ASD-W break//*N* = 37	ASD-WO *N* = 74	*z* =	*p* ≤ ^1^	Effect Size *r* = ^2^
**SEQ Total scores**	51 (41–62)	46.5 (39–57)	−1.94	0.026	0.18
**SEQ subsections**					
HY	21 (18–28)	19 (16–23)	−2.49	0.006	0.24
HY-S	11 (8–15)	10 (7–12)	−1.75	0.04	0.17
HY-NS	11 (10–13)	10 (7.25–12)	−2.52	0.006	0.24
HO	29 (23–34)	29 (22–34)	−1.03	NS	
HO-S	8 (5–9)	7 (5–9)	−1.13	NS	
HO-NS	22 (18–24)	20 (17–25.75)	−0.89	NS	
**SSP Total scores**	132 (113–149)	156 (140.25–165.5)	4.5	0.00001	0.43
**SSP subsections**					
Tactile sensitivity	27 (23–31)	30.5 (28–33)	2.99	0.0014	0.28
Taste/Smell sensitivity	9 (7–13)	18 (14–20)	5.42	0.00001	0.515
Movement sensitivity	13 (10–15)	15 (11.25–15)	1.87	0.03	0.18
Under-responsive/Seeks sensation	21 (17–22)	24.5 (19.25–27.75)	3.43	0.0003	0.33
Auditory filtering	16 (14–22)	22 (20–24)	3.79	0.00008	0.36
Low energy/Weak	26 (20–28)	28 (25.25–30)	2.77	0.028	0.26
Visual/Auditory sensitivity	20 (17–22)	21 (18–23)	1.004	NS	

ASD = Autism Spectrum Disorder; ASD-W = ASD With feeding problems; ASD-WO = ASD WithOut feeding problems; SEQ = Sensory Experience Questionnaire; HY = Hyperresponsiveness; HY-S = HY social items; HY-NS = HY non-social items; HO = Hyporesponsiveness; HO-S = HO social items; HO-NS = HO non-social items; SSP = Short Sensory Profile; NS = Not significant; **^1^** Mann Whitney’s U Test; **^2^** Effect size calculated by using the formula: *r* = *z*/√*N*.

**Table 3:** SSP performances from the whole ASD sample and from each of the ASD-W and ASD-WO subgroups (expressed as percentages of children); statistically significant differences between ASD-W and ASD-WO subgroups; percentages of children as reported in other studies.

SSP Performance CategoriesTitle	All ASD (*N* = 111)	ASD-W (*N* = 37)	ASD-WO (*N* = 74)	ASD-W vs. ASD-WO *p* ≤ ^1^	Cramer’s V Effect Size	Tomchek and Dunn, 2007 [3]	Nadon et al., 2011 [29]	AL-Heizan et al., 2015 [32]
**SSP Total scores**								
Definite Difference	43.2	73	28.4	0.00001 ^3^	0.46	83.6	65.3 ^2^	84.8
Probable Difference	15.3	10.8	17.6			11.4	21.1	8.7
Typical Performance	41.4	16.2	54			5	13.7	6.5
**SSP subsections:**								
**Tactile sensitivity**								
Definite Difference	28.8	48.6	19	0.000015 ^4^	0.33	60.9	36.8	60.9
Probable Difference	22.5	21.6	23			18.5	24.2	21.7
Typical Performance	48.7	29.7	58			20.6	37.9	17.4
**Taste/Smell sensitivity**								
Definite Difference	28.8	62.2	12.2	0.00001 ^5^	0.54	54.1	48.4	52.2
Probable Difference	18.9	16.2	20.2			13.9	15.8	19.6
Typical Performance	52.3	21.6	67.6			32	34.7	28.26
**Movement sensitivity**								
Definite Difference	14.4	24.3	9.5	0.0013 ^6^	0.26	23.1	28.4	50
Probable Difference	17.1	8.1	21.6			21	20	15.2
Typical Derformance	68.5	67.6	68.9			55.9	51.6	34.8
**Under-responsive/Seeks sensation**								
Definite Difference	54.1	78.4	41.9	0.00001 ^7^	0.38	86.1	67.4	89.1
Probable Difference	22.5	13.5	27			7.5	16.8	2.2
Typical Derformance	23.4	8	31.1			6.4	15.8	8.7
**Auditory Filtering**								
Definite Difference	39.6	70.3	24.3	0.00001 ^8^	0.47	77.6	55.8	73.9
Probable Difference	23.5	8.1	31.1			14.6	24.2	8.7
Typical Derformance	36.9	21.6	44.6			7.8	20	17.4
**Low Energy/Weak**								
Definite difference	26.1	37.8	20.2	0.0035 ^9^	0.24	23.1	43.2	58.7
Probable difference	7.2	10.8	5.4			18.9	12.6	10.9
Typical performance	66.7	51.3	74.3			58	44.2	30.4
**Visual/Auditory sensitivity**								
Definite difference	10.8	13.5	9.5	NS		43.8	22.1	34.8
Probable difference	23.4	27	21.6			25.3	31.6	19.6
Typical performance	65.8	59.4	68.9			31	46.3	45.7

ASD = Autism Spectrum Disorder; ASD-W = ASD With feeding problems; ASD-WO = ASD WithOut feeding problems; SSP = Short Sensory Profile; NS = Not significant; ^1^ Chi-square test; ^2^ In this group a mean of 15.5 ± 6 presented with feeding problems; Definite difference vs. Typical performance: ^3^ *p* < 0.00001, Cramer’s V = 0.49; ^4^ *p* < 0.00001, Cramer’s V = 0.38; ^5^ *p* < 0.00001, Cramer’s V = 0.59; ^6^ *p* < 0.00001, Cramer’s V = 0.18; ^7^ *p* < 0.00001, Cramer’s V = 0.38; ^8^ *p* < 0.0018, Cramer’s V = 0.415; ^9^ *p* < 0.0017, Cramer’s V = 0.23.
